# Evaluation of the Ultrastructural Effects on Conjunctival Epithelial Cells of a New Multiple-Action Artificial Tear Containing Cross-Linked Hyaluronic Acid, Cationic Liposomes, and Trehalose with Transmission Electron Microscopy: A Pilot Study

**DOI:** 10.3390/life15101611

**Published:** 2025-10-16

**Authors:** Salvatore Del Prete, Daniela Marasco, Salvatore Troisi, Mario Troisi, Antonio Del Prete

**Affiliations:** 1 Service Biotech s.r.l., 80121 Naples, Italy; danielamarasco.servicebiotech@gmail.com; 2 Ophthalmologic Unit, Salerno Hospital University, 84100 Salerno, Italy; salvatore.troisi@gmail.com (S.T.); troisi165@gmail.com (M.T.); 3 Eye Clinic, Department of Neurosciences, Reproductive and Odontostomatological Sciences, University of Naples Federico II, 80131 Naples, Italy; andelp65@gmail.com

**Keywords:** artificial tears, cross-linked hyaluronic acid, cationic liposomes, trehalose, microvilli, glycocalyx, ocular conjunctiva, transmission electron microscopy (TEM), dry eye disease

## Abstract

**Background:** This study aimed to evaluate the effects of Trimix^®^ on the microvilli and glycocalyx of ocular conjunctival epithelial cells, assessing drug persistence on the cell surface and its interaction with the glycocalyx. Microvilli, vital indicators of cellular health, are altered in inflammatory or toxic conditions, making their restoration a key therapeutic target. **Method:** Building upon previous scanning electron microscopy work, this investigation utilized Transmission Electron Microscopy (TEM) to delve into the direct interaction between Trimix and the cell membrane, elucidating its role in cellular mechanisms. The research involved both an in vitro phase, examining the drug’s molecular arrangement, and an in vivo phase, treating three subjects (healthy, moderate inflammation, severe dry eye) for 30 days. Cytological samples were taken via impression cytology for TEM analysis to observe the drug’s long-term action and its influence on microvillar structures, glycocalyx, and vesicular transport. **Results:** We demonstrated that Trimix stimulated vesicular transport and promoted the formation of a rudimentary glycocalyx, significantly increasing its presence and the number of microvilli in treated patients across all inflammatory grades, even in severe dry eye. **Conclusions:** In conclusion, Trimix acts as an effective glycocalyx substitute, restoring the second mucosal system (SMS) and enabling distressed cells to resume essential exchange functions, offering a novel therapeutic approach for dry eye disease.

## 1. Introduction

**Figure 1:**
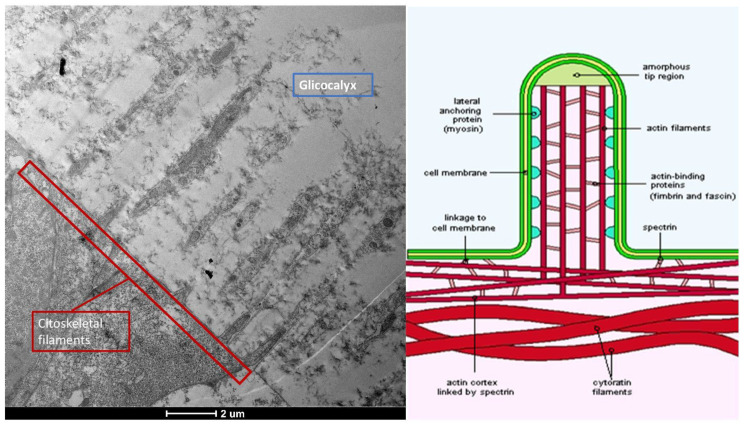
Microvilli are protrusions of the plasma membrane. They consist of a central part with 30–40 actin filaments, which form a bundle bound together by fascin and fimbrin in cross-links. Mag: 2000×; scale bar: 2 mm.

**Figure 2:**
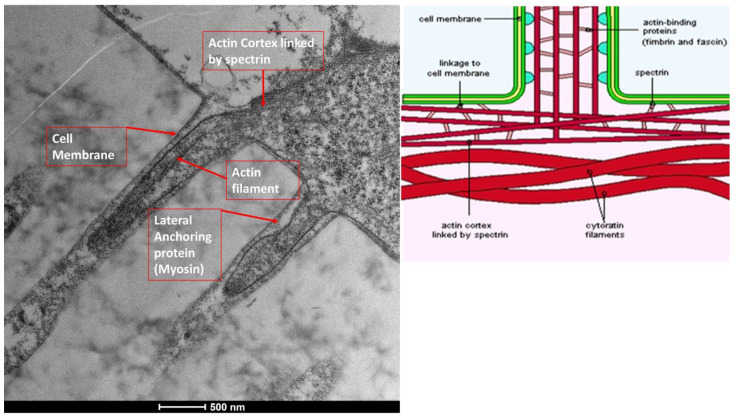
Microvilli are protrusions of the plasma membrane. They move outwards due to lateral anchoring proteins, such as myosin. Mag: 50,000×; scale bar: 500 nm.

**Figure 3:**
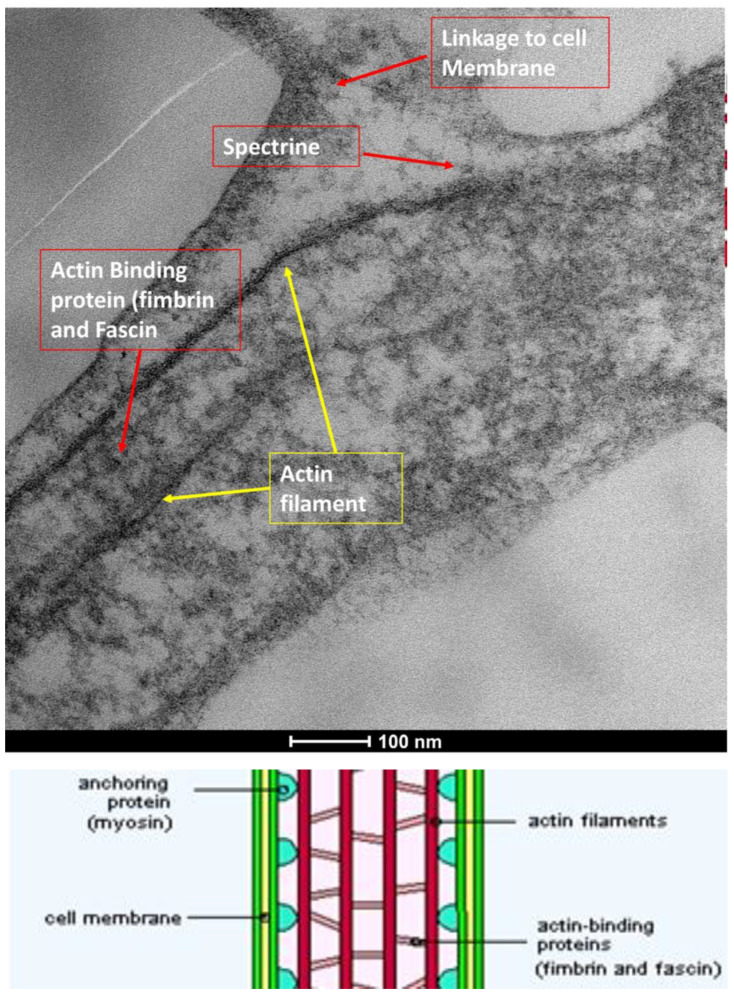
Microvilli are protrusions of the plasma membrane. The plasma membrane covering the microvilli has significant ATPase activity, but it is not solely a communication port with the extracellular phase. Mag: 100,000×; scale bar: 100 nm.

**Figure 4:**
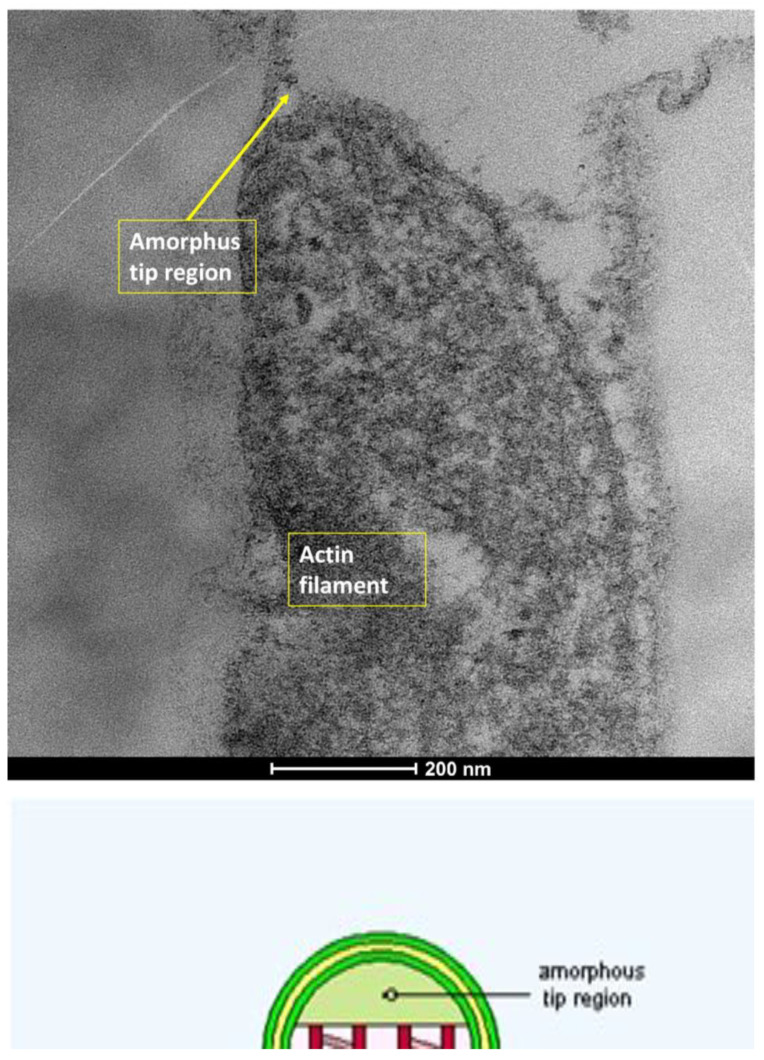
Microvilli are protrusions of the plasma membrane. They consist of a central part with 30–40 actin filaments, which form a bundle bound together by fascin and fimbrin in cross-links. The apical part is formed by a cap of alpha-actinin. Mag: 80,000×; scale bar: 200 nm.

**Figure 5:**
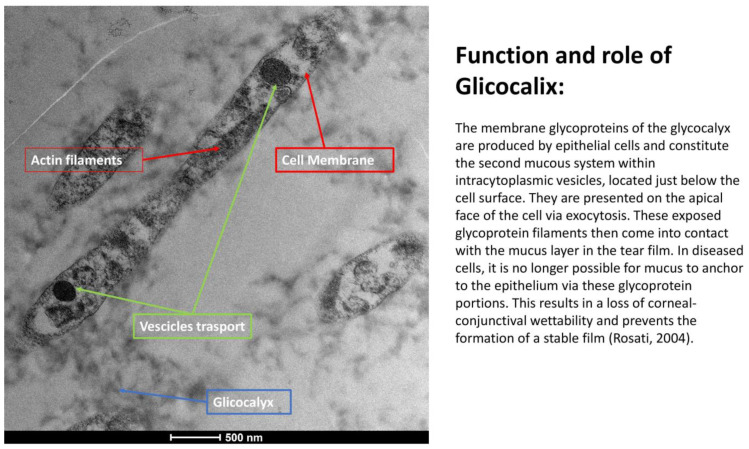
Function and role of the glycocalyx: The membrane glycoproteins of the glycocalyx are produced by epithelial cells and constitute the second mucosal system within intracytoplasmic vesicles, located just below the cell surface. They are presented on the apical face of the cell via exocytosis. These exposed glycoprotein filaments then come into contact with the mucus layer in the tear film. In diseased cells, it is no longer possible for mucus to anchor to the epithelium via these glycoprotein portions. This results in a loss of corneo-conjunctival wettability and prevents the formation of a stable tear film (Rosati [5]). (Mag: 50,000×; scale bar: 500 nm).

The ocular surface, a critical interface between the eye and its environment, relies on the integrity and functionality of its epithelial cells for maintaining health and vision. A key indicator of cellular vitality and function in mucosal tissues, including the ocular conjunctiva, is the presence and morphology of microvilli [1,2]. These plasma membrane protrusions (Figure 1) are dynamic structures (Figure 2) whose alteration often signals cellular distress, whether due to inflammatory states, toxic conditions, or other pathological influences [3]. Understanding the degree of microvillar (Figure 3) alteration is therefore significant in assessing the impact of such conditions on cellular functionality (Figure 4) and, conversely, in evaluating the protective or curative potential of therapeutic agents at a cellular level, specifically their ability to reactivate cellular functions and restore microvilli [4,5]. Dry eye syndrome (DED) is a complex multifactorial condition characterized by an interaction between abnormalities in the tear film, inflammation [6] of the ocular surface, and damage to the mucosal system, which includes the glycocalyx (Figure 5). The glycocalyx, an intricate network of glycoproteins and proteoglycans that coats the surface of epithelial cells, is essential for maintaining ocular surface integrity, tear film stability, and barrier and lubrication functions [7,8]. Alterations or loss of the glycocalyx are well documented in DED and contribute significantly to the pathogenesis of the disease [9,10].

**Figure 6:**
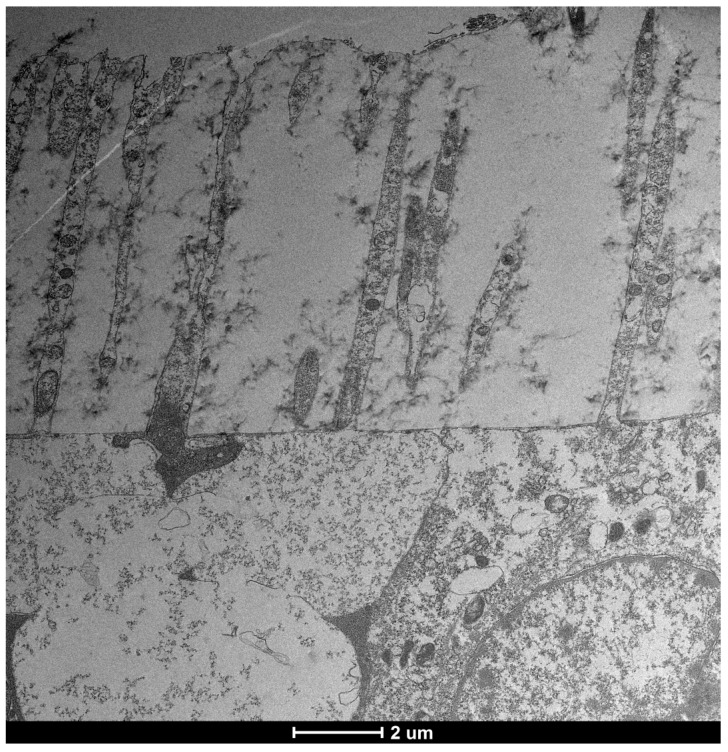
Normal vesicular transport, slightly reduced glycocalyx (but still present), and microvilli of normal length. Mag: 2000×; scale bar: 2 μm.

A crucial component of the ocular surface is the glycocalyx (Figure 6), a filamentous coating found on the apical membrane portion of conjunctival and corneal epithelial cells, particularly at the microvilli and microfolds [11]. This “blanket-like” layer, appearing fluffy under TEM, possesses marked adhesive properties and is fundamental to ocular surface health [12]. The glycocalyx primarily consists of membrane mucins, or “mucin-like” glycoproteins [13], which are O-glycosylated and rich in serine, threonine, and proline. These glycoproteins, with a molecular weight of approximately 200 kDa, are less glycosylated than secreted mucins and feature a peptide chain composed of 20 repeated amino acids with transmembrane regions. Additionally, the glycocalyx is composed of glucidic portions of structural membrane molecules, oligosaccharide branches of glycoproteins and glycolipids, and long polysaccharide chains of membrane proteoglycans. Other components, such as mucins secreted by goblet cells, are adsorbed to the cell surface and contribute to the extracellular environment. These secreted mucins are high-molecular-weight polymers with sulfide bridge interconnections, crucial for forming a stable tear film. The epithelial cells produce glycocalyx membrane glycoproteins, which are presented on the apical face of the cell via exocytosis, forming the “Second Mucosal System” and making contact with the mucus layer in the tear film. In diseased states, such as dry eye disease—recognized as an inflammatory ocular condition—the impaired anchorage of mucus to the epithelium, dependent on these glycoprotein portions, leads to a loss of corneo-conjunctival wettability and prevents the formation of a stable tear film [13,14].

**Figure 7:**
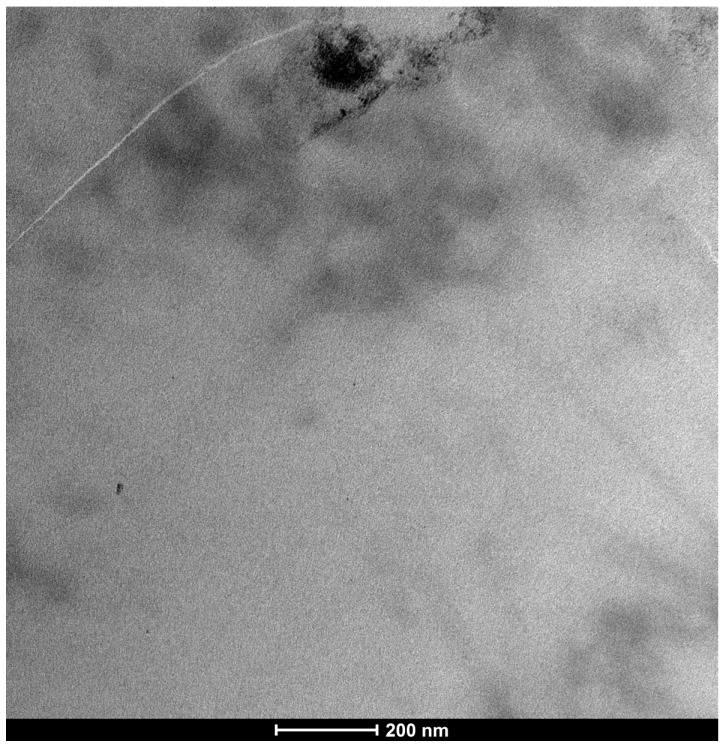
In this image, the drug is identified as an electron-dense substance. It has a specific structure and is significantly thickened. It should be emphasized that the image is a two-dimensional representation of a molecule that develops its shape in three-dimensional space. Therefore, its distribution is affected by the flattening of the image and cannot be compared with images obtained by crystallography, which are more precise. Mag: 80,000×; scale bar: 200 nm.

**Figure 8:**
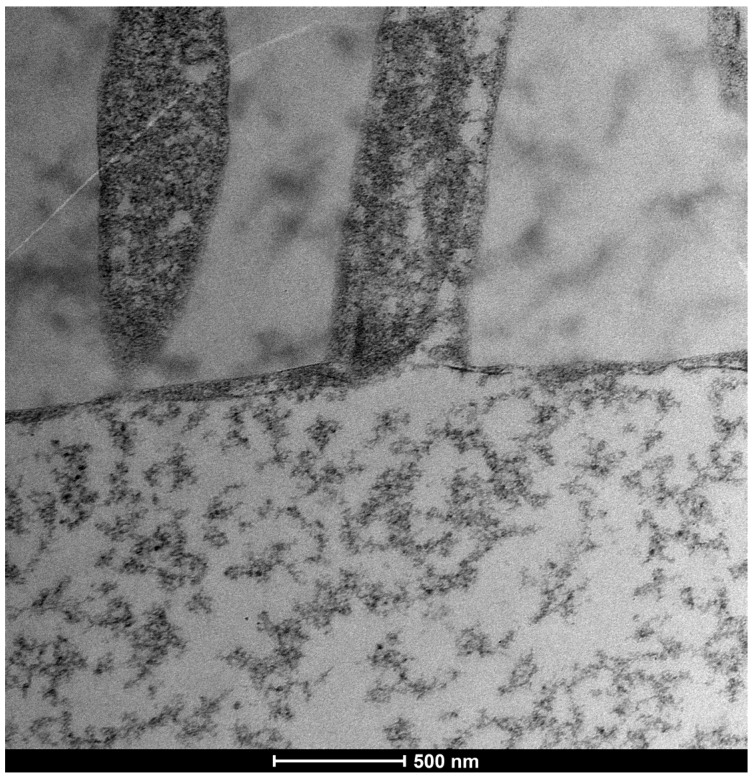
Slight reduction in actin filaments and actin cortex, slight reduction also in cytoskeletal bundles afferent to the base of microvilli protrusions. Vesicular traffic appears normal, as does membrane exchange with the extracellular phase. Mag: 50,000×; scale bar: 500 nm.

This study builds upon previous investigations that utilized Transmission Electron Microscopy (TEM) to evaluate the ultrastructural effects of a new multiple-action artificial tear, including components found in Artificial Tear Containing Cross-Linked Hyaluronic Acid, Cationic Liposomes, and Trehalose (Figure 7), on conjunctival epithelial cells and their interaction (Figure 8) with microvilli [14,15]. While SEM in the previous paper provided valuable surface morphology, the current work advances this research by employing Transmission Electron Microscopy (TEM). TEM allows for a more in-depth investigation into the direct interaction between the Artificial Tear Containing Cross-Linked Hyaluronic Acid, Cationic Liposomes drug and the cellular membrane, probing its role in cells, its interaction with the glycocalyx, and the intricate cellular mechanisms involved in this process [16].

**Figure 9:**
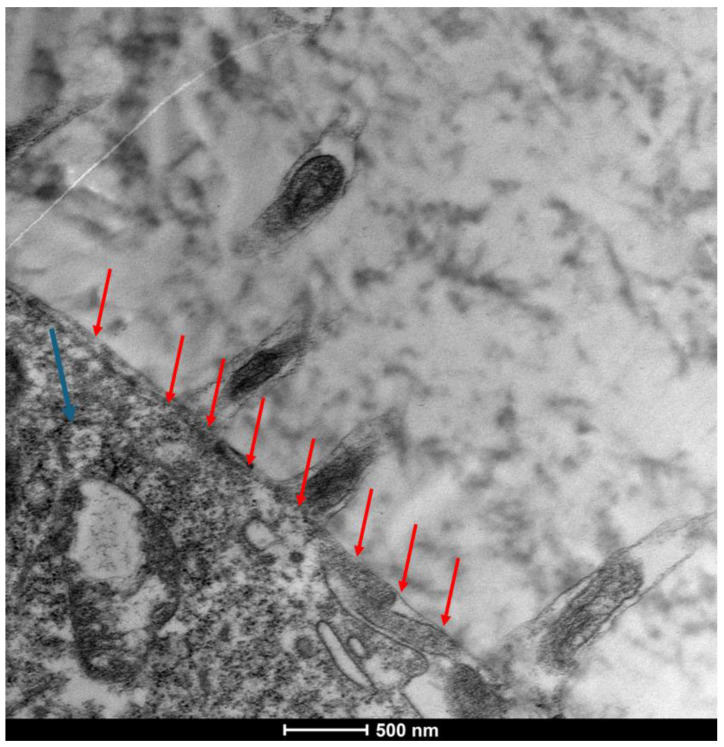
There is a significant presence of the actin cortex and a substantial reduction in microvillus length. There is a moderate presence of the glycocalyx at the base of the microvillus structures and a substantial reduction in plasma membrane thickness, with thickening of the actin fibers that make up the microvillus. There is a phase of contraction and retraction of the protrusions with reduced exchange with the extracellular phase. Flattening of the cellular membrane to the corpus of the cell (red arrows); slight cellular suffering is observed, with a presence of mitochondrial alteration (<blue arrow). Mag: 50,000×; scale bar: 500 nm.

The primary aim of this study is to evaluate the effects of Trimix^®^ on the microvilli of ocular conjunctival epithelial cells and its interaction with the glycocalyx. The in vitro phase consists of the visualization by Transmission Electron Microscopy of the structure of the drug control. The in vivo phase will involve treating three subjects (healthy, moderate inflammation, severe dry eye) with Trimix for 30 days, taking ex vivo tissue samples using impression cytology. TEM will be utilized to assess the drug’s action on microvillar structures and its penetrability (Figure 9) at the cellular level across various inflammatory degrees (grade 0, 1–2, 3–4) (Table 1), verifying any changes in cellular ultrastructure and its interaction with various cellular compartments [17]. Ultimately, this research seeks to elucidate the precise cellular mechanisms through which Trimix exerts its effects, particularly its potential to restore cellular functionality and microvilli, thereby contributing to the understanding of novel therapeutic strategies for ocular surface conditions.

## 2. Materials and Methods

Our investigation employed a dual approach to analyze cellular structures, combining impression cytology for initial specimen collection with ultrastructural analysis for detailed microscopic examination.

For impression cytology, conjunctival epithelium specimens were obtained without the use of anesthetic. This involved gently pressing a piece of cellulose acetate onto the patients’ bulbar upper-temporal conjunctival surface for 3 to 4 s. Following this, the collected cellular material on the cellulose acetate was transferred to an absorbance paper strip by compressing the fragment onto the strip for 30 s, ensuring the cells adhered properly for subsequent steps.

For ultrastructural analysis, samples underwent a meticulous preparation process to preserve their intricate details. Tissues were initially fixed in 2.5% glutaraldehyde within a 0.1 M cacodylate buffer. This was followed by a sequential dehydration process using ethanol, ultimately leading to their embedding in Epon, a standard procedure as detailed by Bonilha et al. [18]. Once prepared, thin sections were cut and subjected to high-resolution imaging. Electron micrographs were acquired using a Tecnai 20 200 kV digital electron microscope (Philips, Hillsboro, OR, USA). Images were transferred in digital format.

### In Vivo Study Design

The study involved the selection of three subjects to be treated with Trimix^®^ (MediVera Compounding Pharmacy, Troy, MI, USA) for 30 days to evaluate the action of the product on microvillar structures at various degrees of ocular surface inflammation according to the Efron grading scale: one healthy patient (grade 0), one with mild/moderate inflammation (grade 2–3), and the other with marked inflammation (grade 4).

The subjects enrolled in the study were 3 (2 men and 1 women), Caucasian, with an age range between 42 and 55 years (mean 50.33 ± 6.02). Exclusion criteria for this study consisted of the following: (I) topical or systemic therapies in the previous 30 days; (II) allergic conjunctivitis; (III) infections and other ocular surface diseases in the last three months; (IV) previous herpetic keratitis; (V) corneal opacities and ulcers; (VI) pregnancy and breastfeeding; (VII) systemic therapies with steroids, immunomodulators, or tetracyclines in the last six months; (VIII) diabetes mellitus; (IX) chronic hepatitis.

At time-0 slit lamp examination (SLE), corneal and conjunctival fluorescein staining were performed according to the Oxford scale (Fluotest). The ocular surface disease index (OSDI) questionnaire was also administered.

## 3. Results

**Figure 10:**
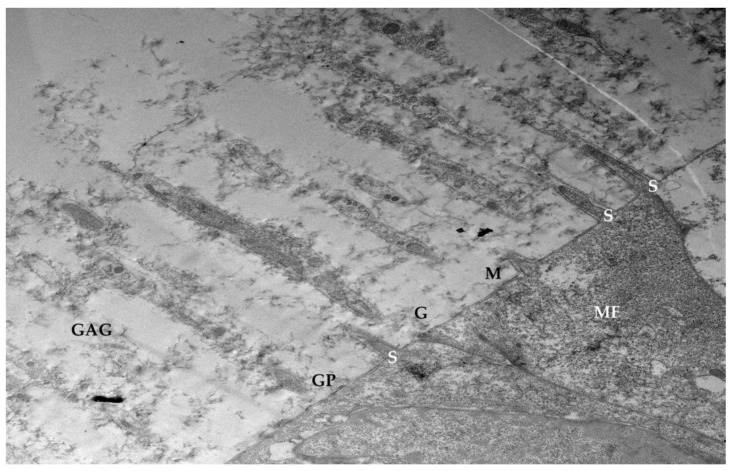
Glycocalyx: Molecular structure of the cell envelope. GAG (glycosaminoglycans); M (cell membrane); GP (glycoproteins embedded in the cell membrane); Ca^2+^ (bivalent calcium cation bridges). Through glycoproteins, the GAGs of the cell envelope are firmly anchored to the cytoskeleton (MF). Spectrin-like molecules (S); oligosaccharide chains of glycoproteins (G); intrinsic non-glycosylated membrane proteins (Rosati 2004 [5]). Mag: 2000×; scale bar: 2 μm.

This study investigated the effects of Trimix^®^ on the microvilli and glycocalyx (Figure 10) of ocular conjunctival epithelial cells using Transmission Electron Microscopy (TEM), both in vitro and in vivo.

### 3.1. Trimix^®^ Structure and Initial Interaction

**Drug TEM Structure:** Trimix^®^ appears as an electron-dense substance in TEM images, exhibiting a specific, significantly thickened morphology (Figure 7). It is important to note that these are two-dimensional representations of a three-dimensional molecule, and therefore, its distribution in the image is affected by flattening.

### 3.2. In Vivo Results: Patient Outcomes at 30 Days

**Figure 11:**
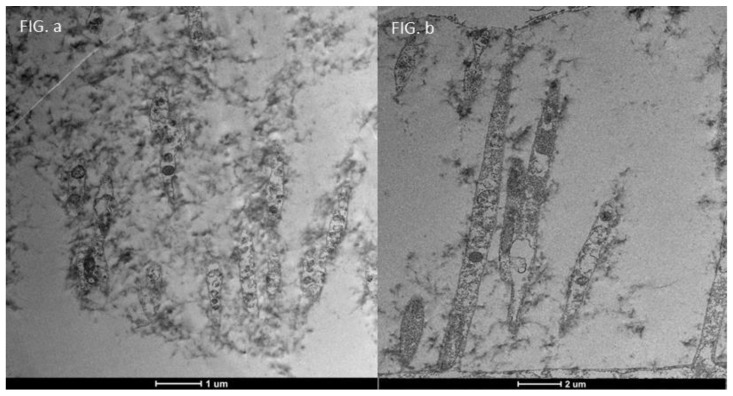
Focus on drug action on tissue; **Healthy patients before and after 30 days of treatment with Trimix**: We can see in (**b**) (Mag. 2000× scale bar 2 mm) that in healthy patients, after 30 days of treatment, the glycocalyx increases ((**a**)—Mag. 5000× scale bar 1 mm) significantly, together with vesicular transport [5] which demonstrates the value of Trimix in the management of even very early stages of cellular distress.

The in vivo phase of the study evaluated the action of Trimix^®^ in three subjects with varying degrees of ocular surface inflammation (healthy, moderate inflammation, severe dry eye) at the time 0 days and after the time 30 days (Figure 11).

### 3.3. Healthy Patients (Grade 0) (Figure 12)

After 30 days of Trimix^®^ treatment, healthy patients exhibited a significant increase in glycocalyx presence and enhanced vesicular transport. This indicates that Trimix^®^ can positively influence even early stages of cellular distress by promoting vital cellular functions (Figure 13).

**Figure 12:**
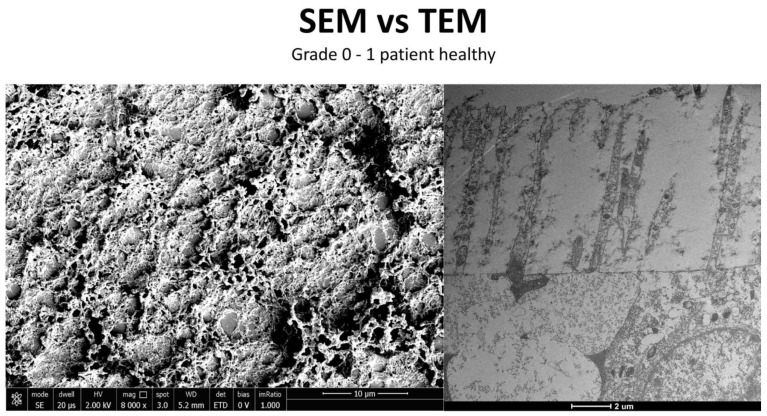
Patient grade 0 alteration of microvilli viewed by SEM (8000×; scale bar: 10 μm) and compared with TEM (2000×; scale bar: 2 μm).

**Figure 13:**
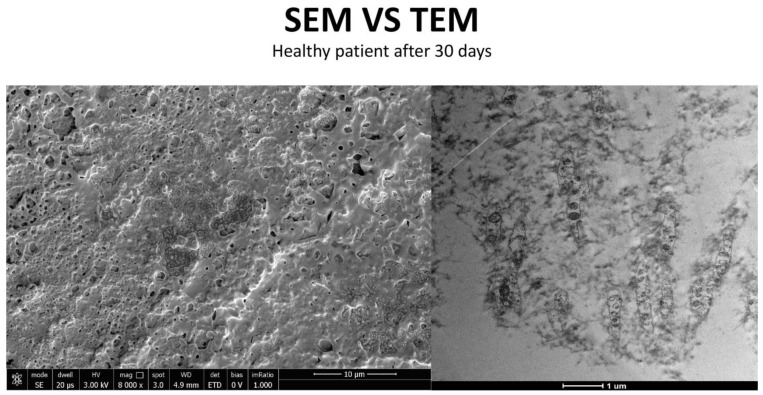
Patient grade 0 alteration of microvilli viewed with SEM (8000×; scale bar: 10 μm) and compared with TEM (5000×; scale bar: 1 μm) after 30 days of treatment.

### 3.4. Moderate Inflammation Patients (Grade 1–2) (Figure 14)

Patients with moderate microvilli alteration (grade 2–3) showed full recovery of cellular functions after 30 days of treatment (Figure 15). This recovery was characterized by:

1. An increase in the number of microvilli.
2. A restoration of the glycocalyx.
3. Improved vesicular traffic.

**Figure 14:**
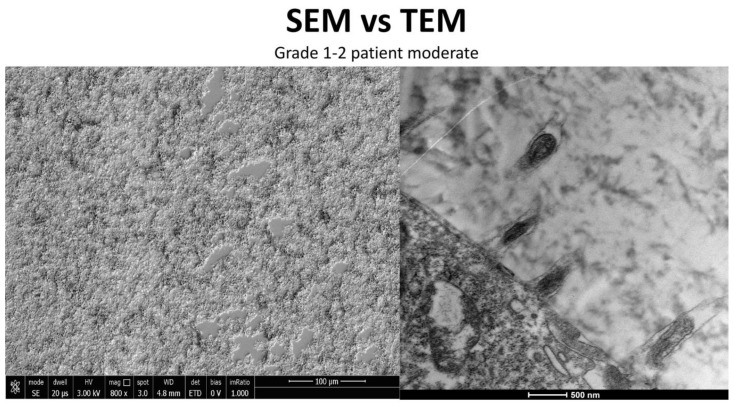
Patient grade 2 alteration of microvilli viewed with SEM (8000× scale bar 10 μm) and compared with TEM (Mag: 50,000×; scale bar: 500 nm).

**Figure 15:**
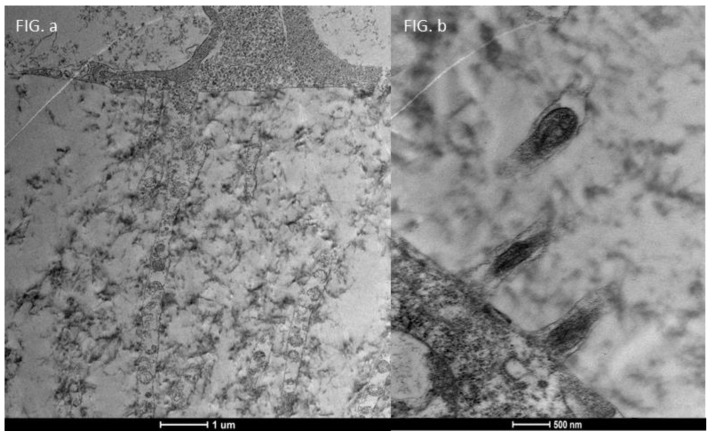
Focus on action of drug trimix: **Moderate patient Before and After**: Compared to the sample examined before treatment ((**b**) Mag. 50,000× scale bar 500 nm), we can observe that in patients with moderate microvilli alteration, i.e., with a grade between 2 and 3, there is full recovery of cellular functions, with an increase in the number of microvilli, glycocalyx and vesicular traffic ((**a**) Mag. 5000× scale bar 1 μm).

**Figure 16:**
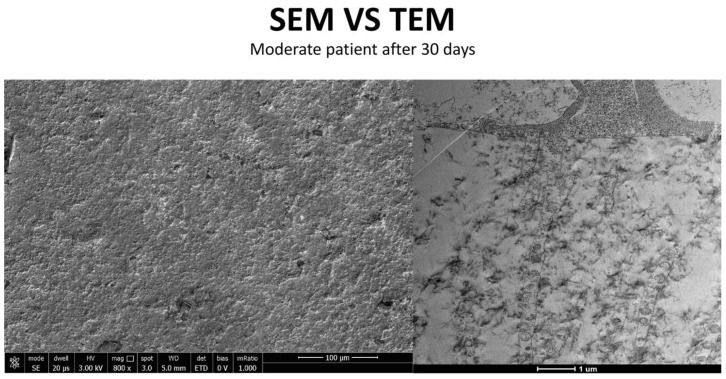
Patient grade 2 alteration of microvilli viewed with SEM (Mag: 8000×; scale bar: 10 μm) and compared with TEM (Mag: 5000×; scale bar: 1 μm) after 30 days of treatment.

This demonstrates Trimix^®^’s ability to reactivate cellular functions and restore microvilli in moderately inflamed conditions (Figure 16).

### 3.5. Severe Dry Eye Patients (Grade 3–4) (Figure 17)

In patients with severe epithelial surface distress (Figure 18), marked by a total absence of microvilli (Figure 19) and a grade 4 alteration, Trimix^®^ treatment resulted in recovery of cellular functions (Figure 20). This included:

1. A slight increase in the number of microvilli.
2. A presence of the glycocalyx.
3. Observable vesicular transport.

**Figure 17:**
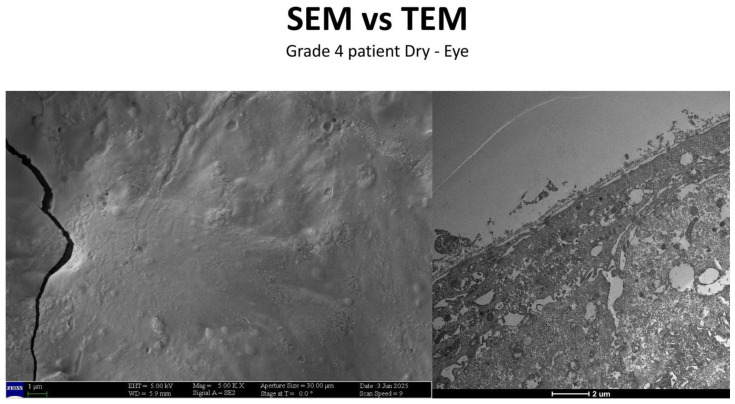
Patient grade 4 alteration of microvilli viewed with SEM (5000×; scale bar: 1 μm) and compared with TEM (2000×; scale bar: 2 μm).

**Figure 18:**
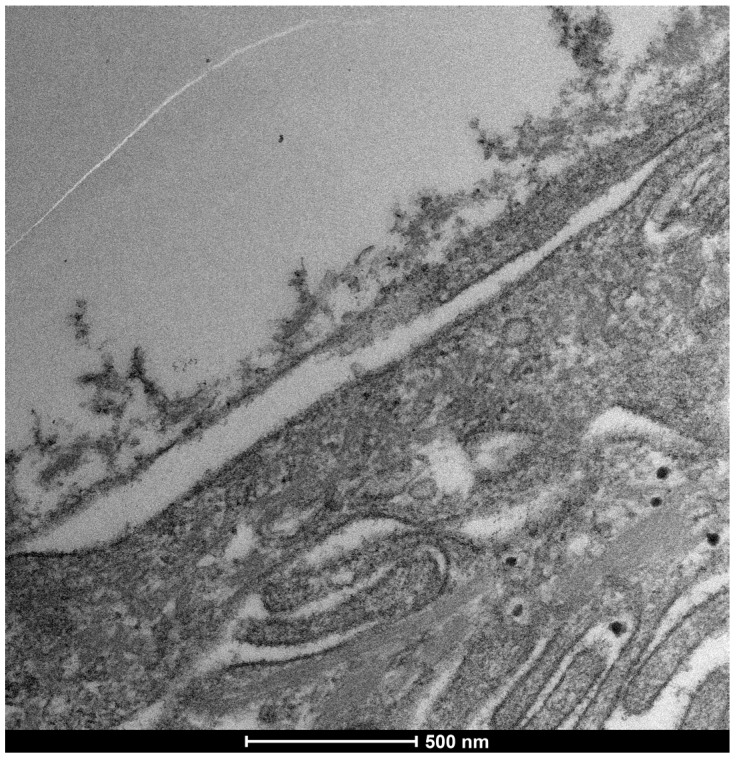
Cells predictive distress parameters: Microvilli. It can be seen here that the activity of the rough endoplasmic reticulum is well preserved, and there are no signs of cellular distress, as can be seen in the photos of patients with mild to moderate alteration and/or reduction in mitochondrial activity. Therefore, the absence of microvilliary structures, as previously described (Del Prete et al. [7]), is the only visible parameter indicating cellular distress. Mag: 50,000×; scale bar: 500 nm.

**Figure 19:**
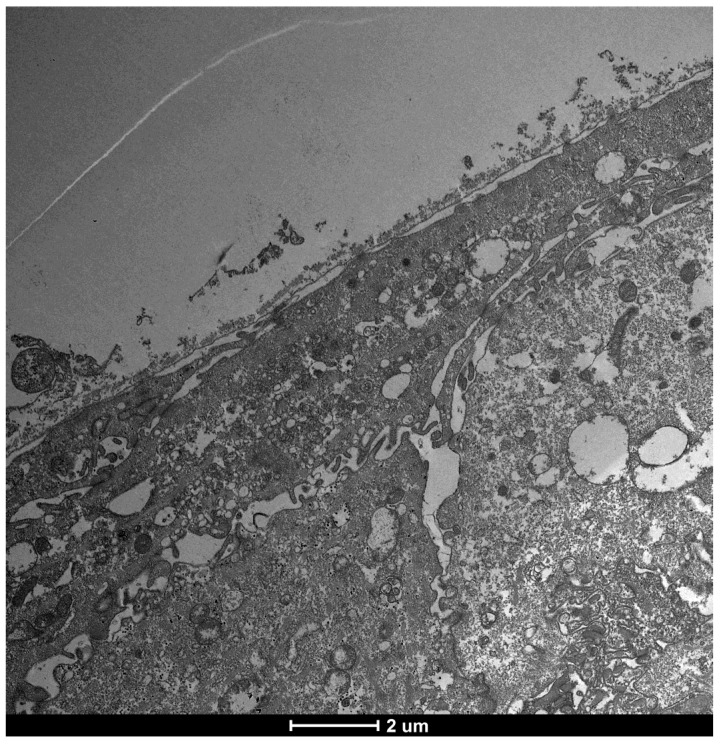
As can be seen in the figure, the microvilli have disappeared entirely and the glycocalyx is absent, although some cellular activity remains. Mag: 2000×; scale bar: 2 μm.

**Figure 20:**
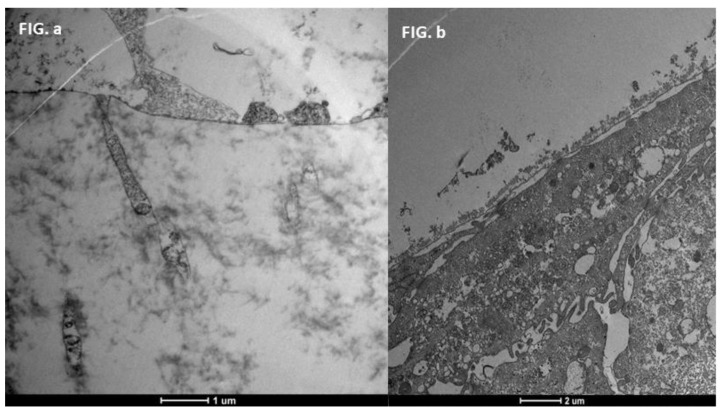
Focus on action of Trimix on patient with severe alteration (grade 4): **Patient with Dry-eye before and After**. In patients with severe epithelial surface distress and therefore a total absence of microvilli and a value of 4 in the table ((**b**) Mag. 2000× scale bar 2 mm), there is a recovery of cellular functions, with a slight increase in both the number of microvilli and the glycocalyx, with the presence of vesicular transport, which is evidence of the health and recovery of the cell and of how trimix, by restructuring the glycocalyx, allows cells to recover their metabolic functions better and more than with other molecules that only protect the cell ((**a**) Mag. 5000× scale bar 1 mm).

**Figure 21:**
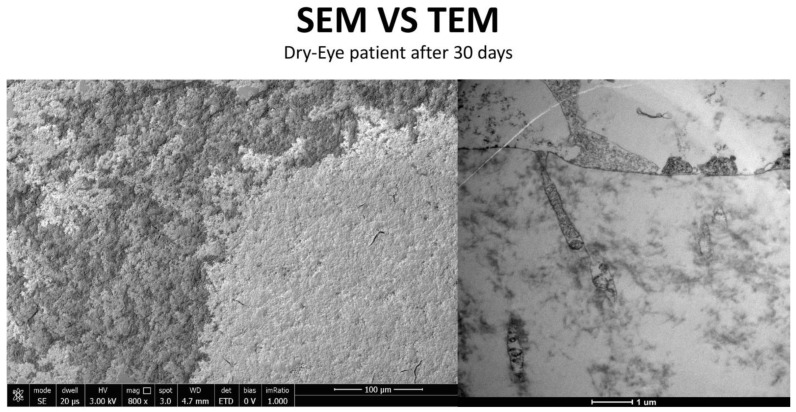
Patient grade 4 alteration of microvilli viewed with SEM (8000×; scale bar: 10 μm) and compared with TEM (5000×; scale bar: 1 μm) after 30 days of treatment.

This suggests that Trimix^®^, by restructuring the glycocalyx, enables severely compromised cells to regain metabolic functions more effectively than other molecules that offer only cellular protection (Figure 21).

### 3.6. Glycocalyx Function and Structure

The glycocalyx, a filamentous coating on the apical membrane of conjunctival and corneal epithelial cells (especially at microvilli and microfolds), exhibits marked adhesive properties.

- Composition: Primarily composed of membrane mucins (O-glycosylated glycoproteins rich in serine, threonine, and proline) and glucidic portions of structural membrane molecules (Figure 1).
- Role in Health: In healthy cells, glycoprotein filaments of the glycocalyx contact the mucus layer in the tear film, ensuring corneo-conjunctival wettability and stable tear film formation.
- Role in Disease: In diseased cells, the impaired anchorage of mucus to the epithelium due to altered glycoprotein portions leads to a loss of wettability and prevents stable tear film formation.

## 4. Discussion

The Artificial Tear Containing Cross-Linked Hyaluronic Acid, Cationic Liposomes, and Trehalose eye drops, a multi-component formulation combining hyaluronic acid, trehalose, and cationic liposomes [19], have demonstrated significant efficacy in alleviating both objective signs and subjective symptoms in patients with mild to moderate DED [20]. Objective improvements include increased tear meniscus height (TMH), non-invasive tear film break-up time (NIBUT), and lipid layer thickness (LLT). Subjective relief is evidenced by a significant reduction in the SPEED (Standard Patient Evaluation of Eye Dryness) score. The favorable tolerability profile of Artificial Tear Containing Cross-Linked Hyaluronic Acid, Cationic Liposomes, and Trehalose further supports its clinical utility [21].

It is essential to emphasize that subjects treated with Artificial Tear Containing Cross-Linked Hyaluronic Acid, Cationic Liposomes, and Trehalose must not have any existing infectious conditions. In such cases, the alteration is no longer linked to cellular suffering or to the alteration of the balance between the cell and the pre-corneal film but is solely related to the toxic and damaging activity of pathogens. Syndromes such as DED [22] can also promote colonization by external pathogens; therefore, an assessment of the patient’s infectious status is necessary before administering cell-revitalizing eye drops, so as not to compromise the therapeutic approach.

Study results indicate that Artificial Tear Containing Cross-Linked Hyaluronic Acid, Cationic Liposomes, and Trehalose acts as a valid substitute and restorer of the glycocalyx, an integral part of the Second Mucosal System (SMS) [23]. Unlike other molecules, such as hyaluronic acid alone, the advantage of Artificial Tear Containing Cross-Linked Hyaluronic Acid, Cationic Liposomes, and Trehalose lies in its ability to actively rebuild the glycocalyx [10]. This rebuilding action is particularly significant, going beyond the simple cellular revitalization observed with molecules such as hyaluronic acid [24,25].

The multi-component formulation of Artificial Tear Containing Cross-Linked Hyaluronic Acid, Cationic Liposomes, and Trehalose is consistent with the current therapeutic approach to DED, which often requires a multi-pronged intervention to address the complexity of the disease [26,27]. Hyaluronic acid is known for its moisturizing and viscoelastic properties, which prolong its residence time on the ocular surface [28,29,30]. Trehalose protects cells from osmotic stress by stabilizing proteins and cell membranes [31]. Cationic liposomes, on the other hand, improve the stabilization of the lipid layer of the tear film and facilitate the release of active ingredients onto the ocular surface, also contributing to greater adhesion and prolonging the time spent on the ocular surface [32,33].

### Mechanism of Action and Future Prospects

As observed in various groups of patients already treated, Artificial Tear Containing Cross-Linked Hyaluronic Acid, Cationic Liposomes, and Trehalose allows distressed cells to resume normal exchange functions, which are crucial for the health and functionality of the conjunctival and corneal mucosa [34]. By restructuring the glycocalyx, Artificial Tear Containing Cross-Linked Hyaluronic Acid, Cationic Liposomes, and Trehalose allows cells to recover their metabolic functions and re-establish essential exchanges with the extracellular environment. Its mechanism of action, which addresses multiple aspects of the pathophysiology of DED, including hydration, epithelial protection, and lipid layer stabilization, aligns with the complex nature of the disease. This offers a new perspective on the therapeutic approach for patients with dry eye syndrome, positioning Trimix^®^ as a pioneering molecule in its treatment [35].

However, the claim that Artificial Tear Containing Cross-Linked Hyaluronic Acid, Cationic Liposomes, and Trehalose is a “pioneering” treatment for DED [36] through the restoration of the glycocalyx requires further analysis. Although the logic of acting directly on the glycocalyx [37,38,39] is scientifically valid and promising, it is essential to evaluate the strength of the clinical evidence demonstrating the actual reconstruction of the human glycocalyx [40,41,42,43,44,45] in vivo and the extent of this restoration compared to other treatments [46,47,48,49]. Transmission electron microscopy (TEM), mentioned in previous discussions, is a valuable tool for the ultrastructural analysis of the glycocalyx, but direct data on Artificial Tear Containing Cross-Linked Hyaluronic Acid, Cationic Liposomes, and Trehalose’s ability [50] to induce significant glycocalyx regeneration in large-scale clinical studies would be of great value. Small studies, like the pilot study presented here, can provide high-quality results quickly; these studies provide data that should be used to design larger confirmatory studies. The goal, therefore, is to provide reliable evidence that will support subsequent studies [51].

## 5. Conclusions

The ocular conjunctival interface, with its complex microvilli and vital glycocalyx, is crucial for maintaining tear film stability, ensuring hydration and lubrication, and acting as a robust protective barrier for the eye. Transmission electron microscopy (TEM) has played a key role in revealing significant ultrastructural alterations of these components in Dry Eye Disease (DED). These alterations include a reduction in the density and height of microvilli, together with the absence or thinning of the glycocalyx of the ocular surface. These profound changes at the cellular level directly compromise tear film dynamics and the overall health of the ocular surface. In this context, Trimix presents itself as a valid substitute for the glycocalyx; more precisely, it serves to restore the SMS layer. Compared to other molecules, such as hyaluronic acid alone, the advantage of Trimix lies in its steric hindrance, which prevents the internalization of the drug. Its therapeutic role is entirely external, focused on the reconstruction of the glycocalyx. As observed, Trimix allows damaged cells to resume normal exchange functions, which are essential for the conjunctival and corneal mucosa. The restoration of the glycocalyx for therapeutic purposes is evidenced by the various patients treated. This creates a new vision for the therapeutic approach to patients with dry eye, where Trimix stands out as a pioneering molecule in the treatment of DED. The detailed ultrastructural insights provided by TEM highlight the profound cellular damage that occurs in DED. This shifts the understanding of the disease from a simplistic view of tear deficiency to one of complex epithelial and glycocalyx dysfunction. The proven success of multicomponent therapies such as Trimix^®^ highlights the need for comprehensive approaches that target the various interconnected pathological pathways in DED, rather than focusing on isolated symptoms. The dynamic nature of the glycocalyx and its susceptibility to disease-induced changes suggest its considerable potential as a biomarker for the diagnosis of DED, for monitoring disease progression, and as a direct therapeutic target. This suggests a potential paradigm shift in the development of DED treatment, moving towards therapies specifically designed to protect, restore, or mimic the functions of the glycocalyx.

## Figures and Tables

**Table 1:** The table presents the classification of microvillus alteration as a predictive mechanism (Del Prete et al. [7]) of alteration in the stability of the precorneal film. Integration with TEM data highlights a more refined mechanism directly related to the glycocalyx, which is responsible for the fine regulation mechanisms of the precorneal film. This integration reinforces and amplifies the predictive capacity of microvillus analysis.

SEM Evaluation Table				
**Grade 0**	**Grade 1**	**Grade 2**	**Grade 3**	**Grade 4**
Microvilli on site	Microvilli on site	Microvilli on site	Microvilli on site	Microvilli not on site smooth area
Normal Surface	Normal Surface	Low alteration of the Surface	High alteration of the Surface	High alteration of surface
High microvilliar distribution	Low microvilliar distribution	microvilliar distribution on spot	microvilliar sensible reduction with spotted smooth areas	No microvillar presence on surface area reading
Structure of Microvilli are arborescent	Structure of Microvilli are not totally arborescent	Pseudo-microvilli structure	Pseudo-microvilli structure	Smooth surface, Moon-surface
**TEM Evaluation Table**				
**Grade 0**	**Grade 1**	**Grade 2**	**Grade 3**	**Grade 4**
Presence of glicocalyx and presence vescicular transport	Reduction of Glicocalyx and presence of vescicular transport	Absence of glicocalyx and reduction of vescicular transport	No glicocalyx, No vescicular transport, Reduction of lenght of microvilli	flattening of the plasma membrane, absence of exchange with the outside

## Data Availability

All data are provided in the main text.

## Abbreviations

The following abbreviations are used in this manuscript:

DED	Dry-Eye Disease
GvHD	Chronic graft-versus-host disease
SPEED	Standard Patient Evaluation of Eye Dryness
SEM	Scanning Electron Microscopy
TMH	Tear meniscus height
NIBUT	Non-invasive tear film break-up time
LLT	Lipid layer thickness
TEM	Transmission Electron Microscopy
SMS	Second Mucosal System
